# Time-Dependent CO_2_-Brine-Rock Interaction Effect on Sand Onset Prediction: A Case Study of Dolomite-Rich Sandstone in Air Benakat Formation, South Sumatra, Indonesia

**DOI:** 10.1021/acsomega.4c09499

**Published:** 2025-04-09

**Authors:** Prasandi Abdul Aziz, Taufan Marhaendrajana, Bagus Endar Bachtiar Nurhandoko, Utjok W. R. Siagian

**Affiliations:** † Department of Petroleum Engineering, Faculty of Mining and Petroleum Engineering, Institut Teknologi Bandung, Jl. Ganesha No. 10, Bandung 40132, Indonesia; ‡ Department of Physics, Faculty of Mathematics and Natural Sciences, Institut Teknologi Bandung, Jl. Ganesha No. 10, Bandung 40132, Indonesia; § Research Center for CO_2_ and Flare Gas Utilization, Institut Teknologi Bandung, Jl. Ganesha No. 10, Bandung 40132, Indonesia; ∥ Rock Fluid Imaging (RFI) Laboratory, Bandung 40124, Indonesia

## Abstract

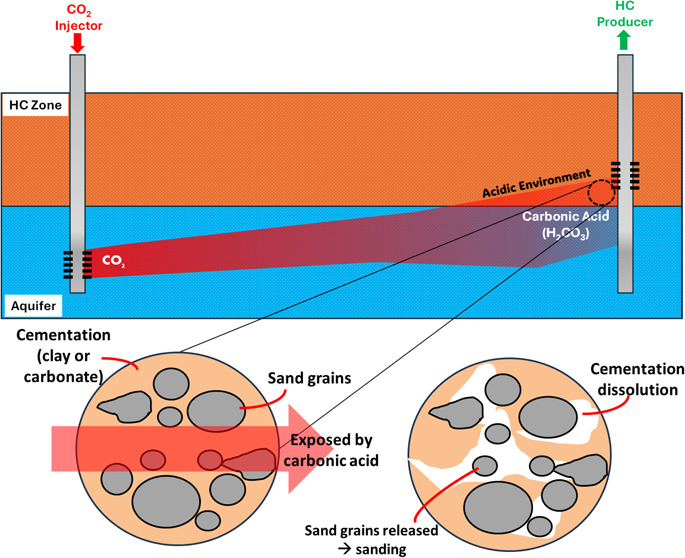

CO_2_ injection into geological formation for carbon capture, utilization, and storage (CCUS) including enhanced oil or gas recovery provides a solution to reduce CO_2_ emissions and bring on potential economic benefits. However, field operation possesses potential challenges both for injection and production wells. Operational constraints commonly considered are weighted around the injection well and related to well and formation integrity (e.g., limiting the risk of injection-induced fracturing). On the other hand, complexities of CO_2_-brine-rock interaction affect sand production phenomena in production wells. This study investigates CO_2_-brine-rock interaction to reflect CO_2_ injection impact toward reservoir fluid and rocks. This study involves extensive experimental works, namely, time-lapse dry mass measurements, brine compositions and pH analysis, X-ray diffraction (XRD), scanning electron microscopy–energy-dispersive spectroscopy (SEM-EDS), petrographic thin section analysis, porosity measurements, and elastic wave velocity measurements. CO_2_-brine-rock batch experiment was designed and utilized to observe mineral dissolution, pore structure alteration, as well as rock physics alteration caused by CO_2_-brine-rock interactions. An outcrop sample of dolomite-rich sandstone in Air Benakat formation, South Sumatra, Indonesia, was used as a case study. This study shows that dolomite dissolution was observed and led to ∼6.6% porosity improvement as well as rock strength reduction (as shown by ∼4.3% of P wave and ∼6.2% of S wave reduction, respectively). The results of experimental works were then used to construct sand onset prediction model that considers rock strength alteration caused by CO_2_-brine-rock interactions. The sand onset prediction model demonstrates an acceleration of sand onset occurrence due to CO_2_-brine-rock interactions which can assist the operator to design a better sand management strategy in producer wells.

## Introduction

**Figure 1:**
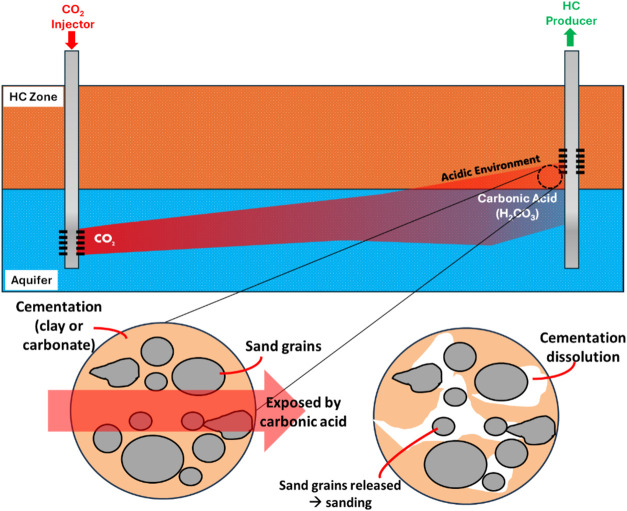
Illustration of cementation dissolution due to CO_2_ injection (e.g., CCUS) operation.

CO_2_ injection into geological storage (e.g., depleted oil and gas reservoirs, CO_2_ enhanced oil or gas recovery, carbon capture, utilization, and storage (CCUS), saline aquifer, coal seams, coal bed methane, basalts, and many others) provides a solution to reduce CO_2_ emissions into the atmosphere.^1^ In Indonesia, the feasibility of CO_2_ injection projects and potentials have been studied to support CO_2_ emissions reduction.^2−7^ The underlying reactions, represented by eqs 1–3, show carbonic acid as a product of CO_2_ that dissolves into water and dissociates H^+^ ions. Carbonic acid is a mild acid with pH ranging from 4 to 5.^8−10^ With the presence of carbonic acid, therefore sand grain might be released due to cementation dissolution, and it can potentially cause sanding phenomena problem in the producer well. Figure 1 shows the illustration of cementation dissolution phenomena near producer well due to CO_2_ injection in aquifer.
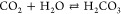
1
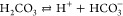
2
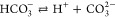
3

Several reported experimental studies investigated the effects of pressure and temperature on CO_2_ solubility in water.^11−13^ Carbonic acid can also react with certain reservoir rock mineral which leads to dissolution. Dreybrodt and co-workers^14,15^ showed the dissolution of calcite with the presence of water and aqueous phase of CO_2_, such as depicted in eq 4. Several authors have investigated the dissolution of dolomite with hydrogen cation as indicated in eq 5.^16,17^ The dissolution of calcite and dolomite releases Ca^2+^ and Mg^2+^ into aqueous/brine solution.^13,16^ The dissolution of quartz can occur with the presence of hydrogen cation (eq 6) as indicated by Crundwell).^18^
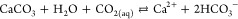
4
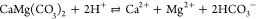
5
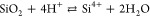
6

**Figure 2:**
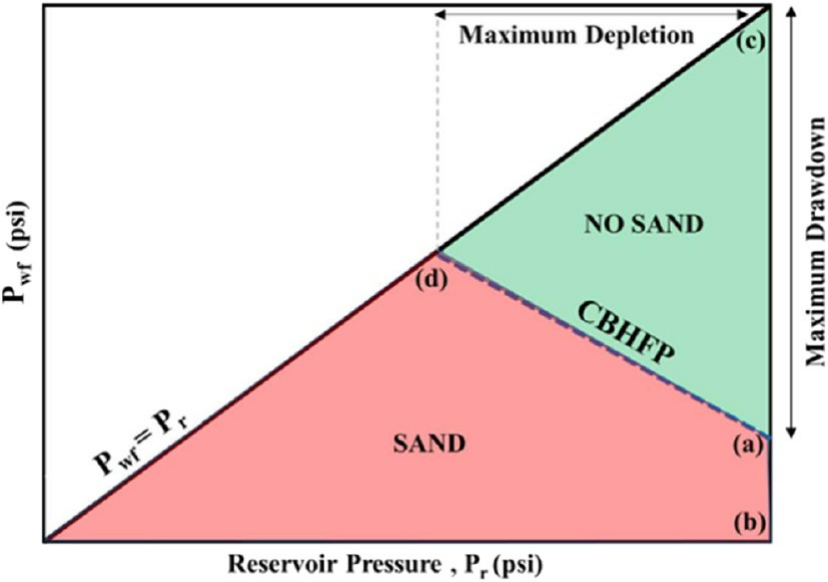
Illustration of CBHFP sand onset criteria. When the *P*_wf_ vs *P*_r_ profile is in the red area, then sanding will likely occur. (a, b) initial value of CBHFP when the production begins; (a–c) maximum drawdown before sanding; (a–d) *P*_r_ is depleting and CBHFP is increasing.

**Figure 3:**
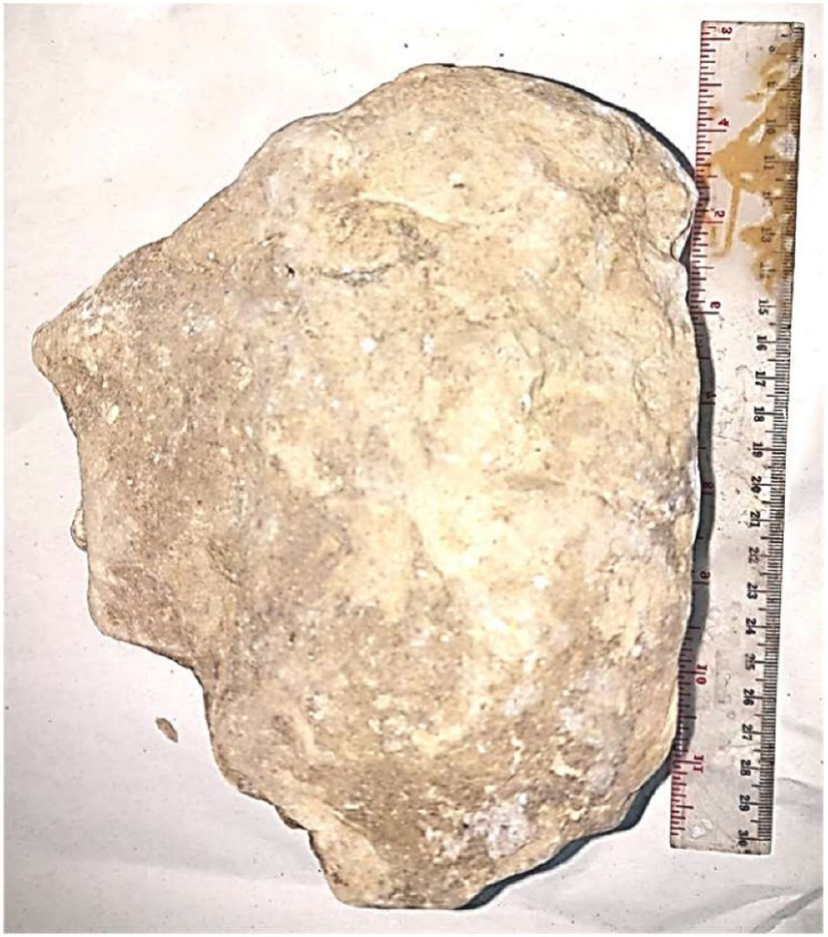
Outcrop samples of Air Benakat Formation (ABF) sandstone.

**Table 1:** Mineralogical Composition of ABF Rock Sample from XRD (Initial)

mineral	chemical formula	concentration (wt %)
dolomite	CaMg(CO_3_)_2_	79
quartz	SiO_2_	17
kaolinite	Al_2_Si_2_O_5_(OH)_4_	4

**Figure 4:**
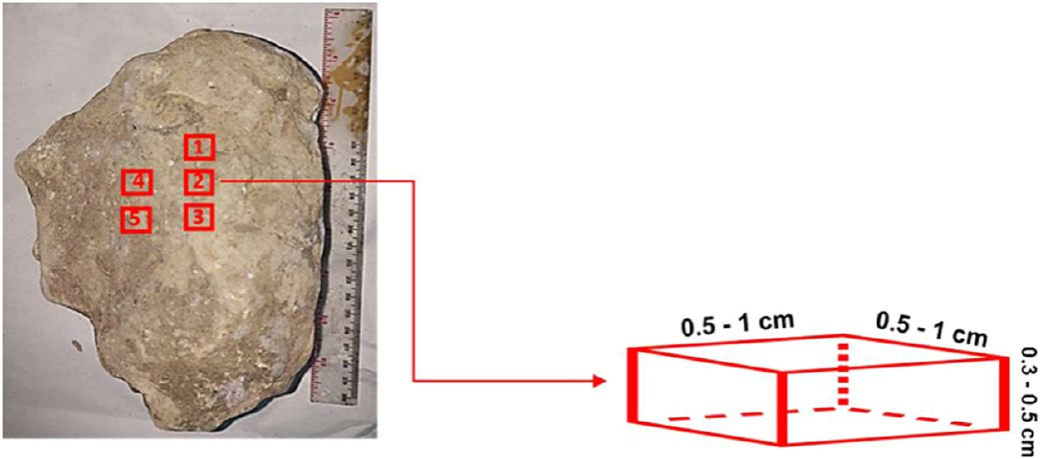
Small cubic samples for XRD, SEM-EDS, and thin section analysis.

**Figure 5:**
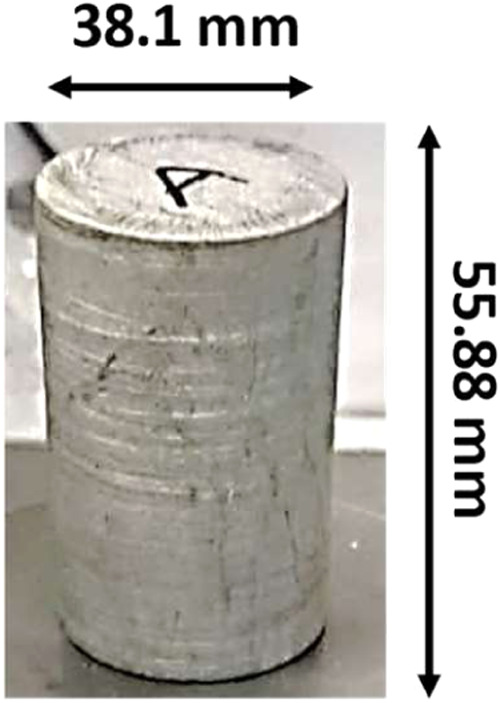
Cylindrical sample for elastic wave velocity measurements.

**Table 2:** Reservoir Conditions of ABF Interval in Analogue Well at a Depth Interval of 895–905 m-TVD (Data from P-3 Final Well Report)

parameter	unit	value
analogue depth interval	m-TVD	895–905
brine salinity	mg/L	15,000 (NaCl)
reservoir or pore pressure *P*_p_	psi	1300
fault regime		Strike-slip
vertical stress *S*_v_	psi	2500
minimum horizontal stress *S*_hmin_	psi	1652
maximum horizontal stress *S*_Hmax_	psi	2610
P wave (*V*_p_)	m/s	2200–2500
S wave (*V*_s_)	m/s	900–1300

**Figure 6:**
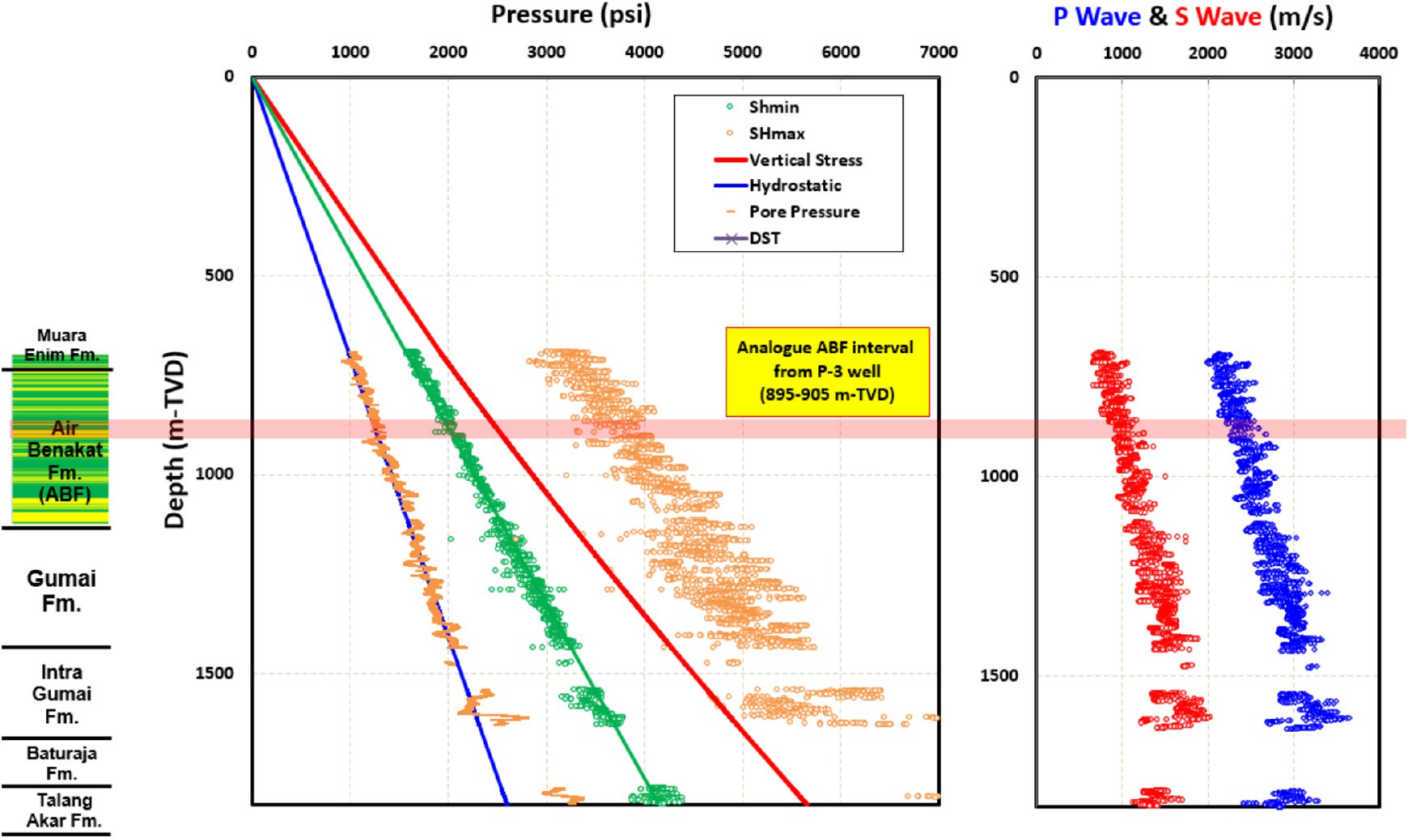
P-3 well profile: pore pressure, S_hmin_, *S*_Hmax_, vertical stress, P wave, and S wave (red shaded area shows analogue ABF depth interval at 895–905 m-TVD).

**Figure 7:**
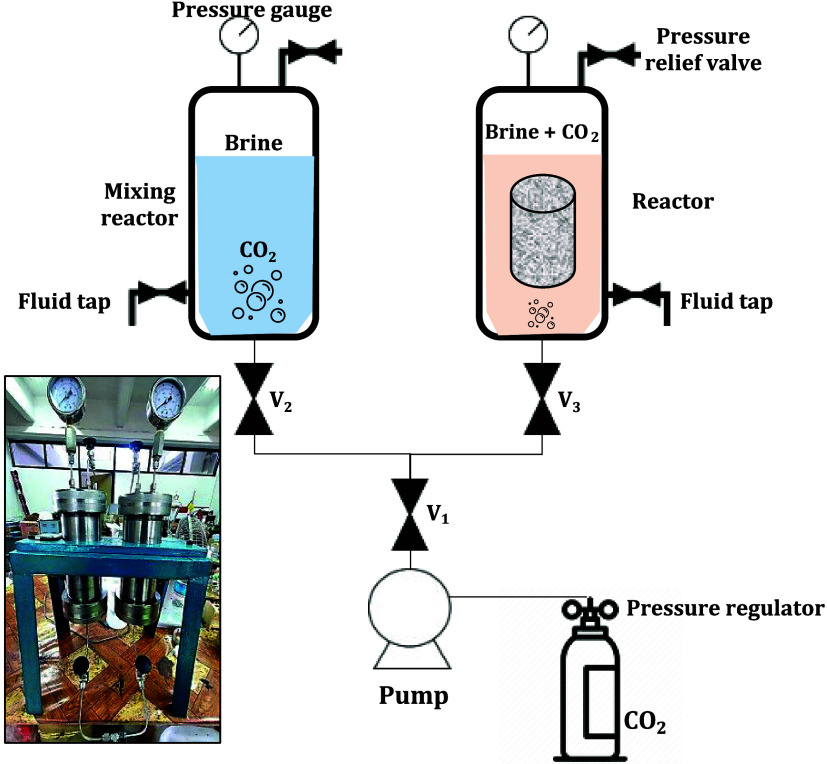
CO_2_–water–rock batch experiment setup and procedure: (1) Enter 1.0 L of water + NaCl solution into the mixer reactor; (2) Close the reactor and make sure there were no leakage; (3) Flow CO_2_ into the mixer reactor (open valves V1 and V2, close V3) until reactor pressure reaching 1300 psi; (4) Maintain pressure in the reactor; (5) Close valves V1 and V2, wait ±24 h to ensure CO_2_ fully saturated into the water + NaCl solution; (6) Flow the solution from the mixer into the soaking reactor containing the rock sample (open valves V2 and V3, close valve V1); (7) After pressure reached 1300 psi, close valves V2 and V3. If necessary, adjust the reactor pressure via pump and valves V1 and V3; (8) Soak the rock samples for 3–7–14 days and record temperature during test periods; (9) After the soaking period is completed, open the pressure relief valve, and then immediately put rock sample in a sealed plastic container (to prevent air contamination).

**Table 3:** Mineral Compositions of Brine: before and after CO_2_ Injection (14 Days)

parameter	unit	methods	before CO_2_ injection	after CO_2_ injection (14 days)
calcium	mg/L	APHA 3500-Ca	24.3	103.0
magnesium	mg/L	APHA 3500-Mg	6.60	7.04
chloride	mg/L	APHA 4500-Cl B	8628	5427
sodium	mg/L	APHA 3111-B	2619	2661
silica	mg/L	APHA 4500-SiO_2_-C	20.3	19.3
aluminum	mg/L	APHA 3113-B	10.8	10.4

**Figure 8:**
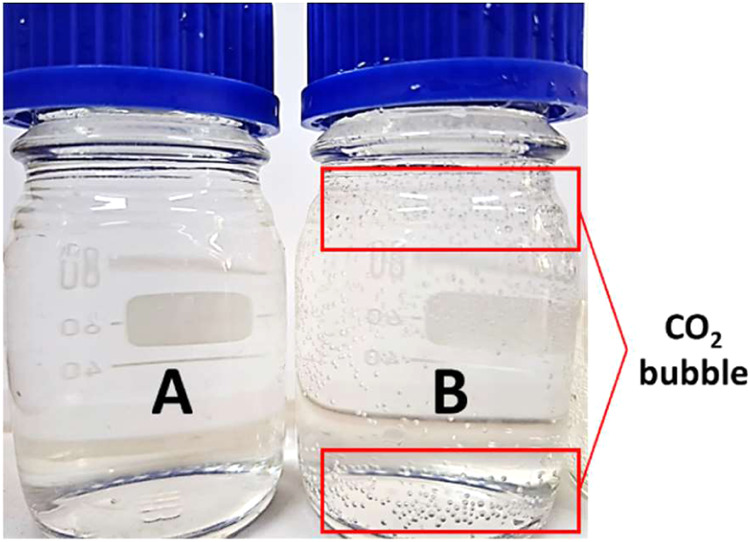
Water analysis: (a) Brine sample (NaCl 15,000 mg/L) before CO_2_ injection; (b) brine sample after CO_2_ injection and used to soak rock sample for 3 days.

**Figure 9:**
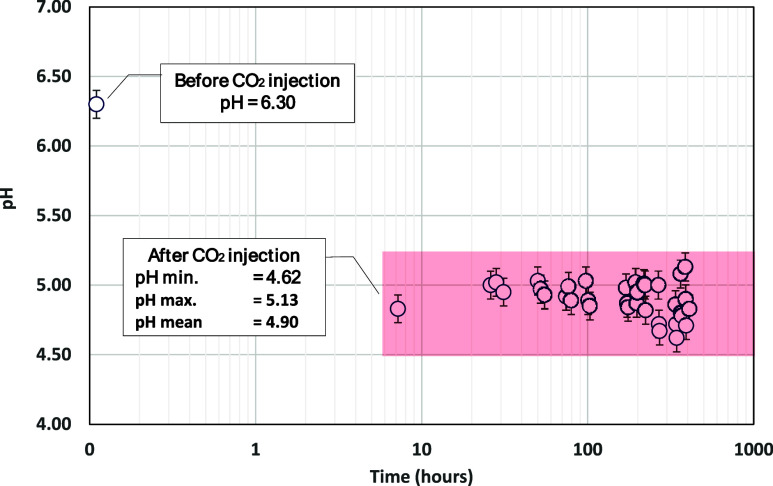
pH of brine (before and after CO_2_ injection) measurements over time.

**Figure 10:**
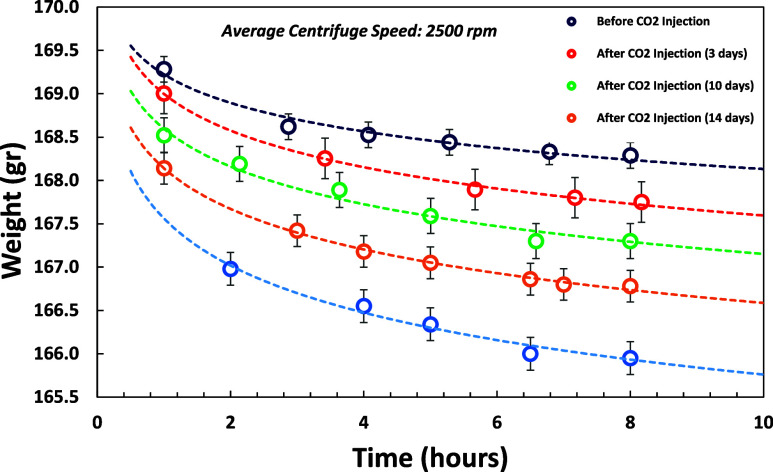
Dry sample (cylindrical) weight measurements after centrifugation with 2500 rpm average speed.

**Figure 11:**
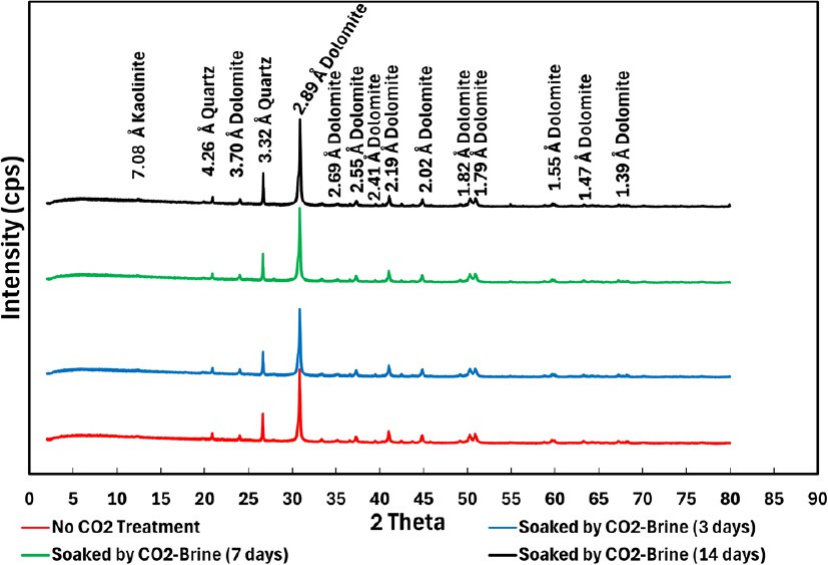
XRD measurements for various CO_2_ soaking times (0–14 days).

**Figure 12:**
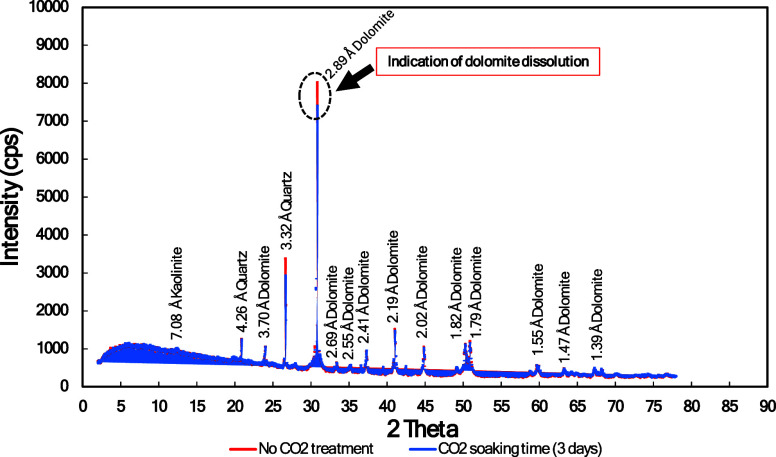
Indication of dolomite dissolution from XRD measurements.

**Table 4:** Mineralogical Composition of ABF Samples after CO_2_ Treatment from XRD

		concentration (wt %)		
mineral	chemical formula	CO_2_ soaking time (3 days) (%)	CO_2_ soaking time (7 days) (%)	CO_2_ soaking time (14 days) (%)
dolomite	CaMg(CO_3_)_2_	82	80	75
quartz	SiO_2_	15	15	21
kaolinite	Al_2_Si_2_O_5_(OH)_4_	3	5	4

**Figure 13:**
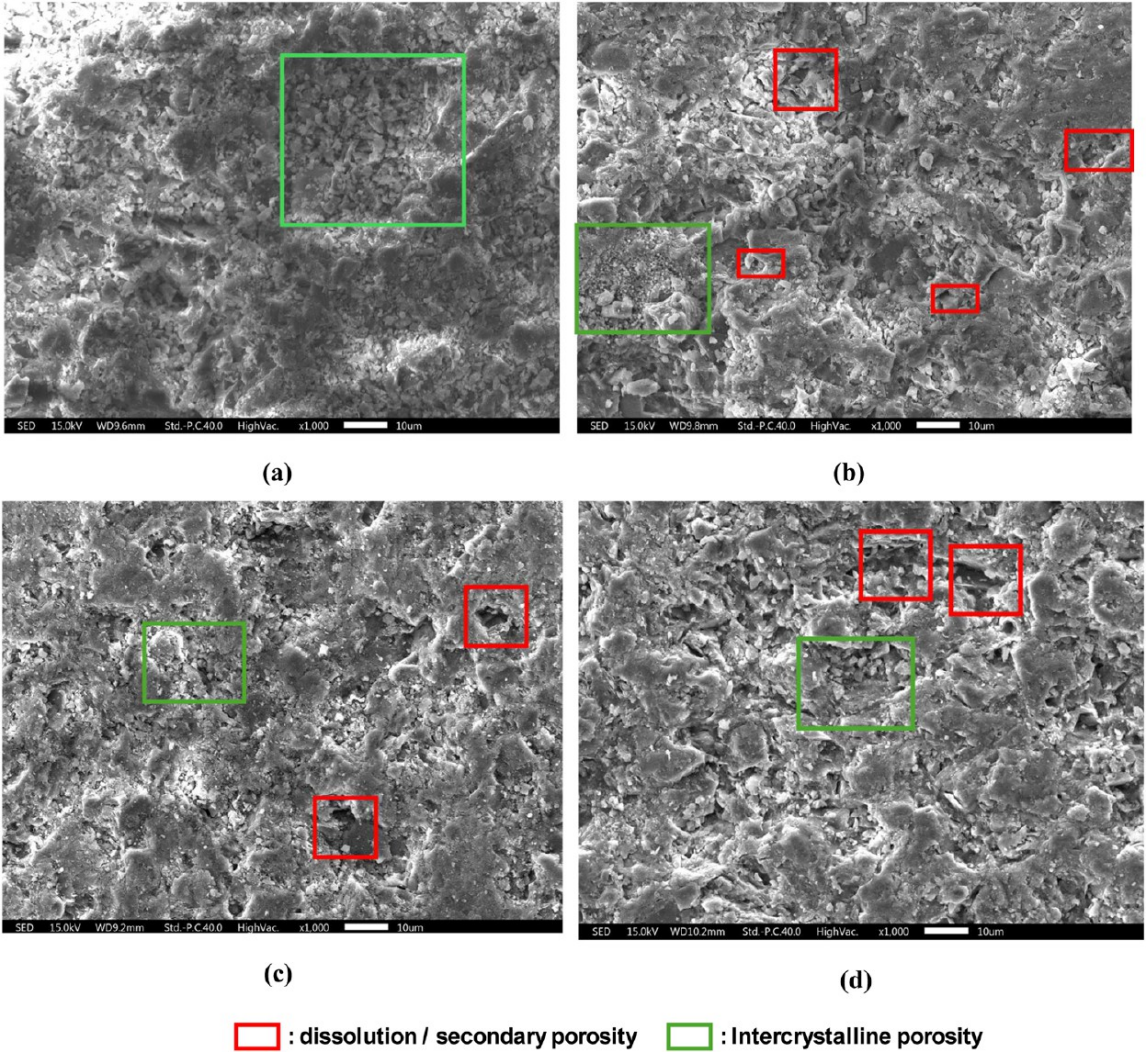
SEM results of ABF rock samples: (a) No CO_2_ treatment; (b) 3 days of CO_2_ soaking; (c) 7 days of CO_2_ soaking; (d) 14 days of CO_2_ soaking.

**Table 5:** Elements Composition of ABF Samples before and after CO_2_ Treatment from SEM-EDS Analysis

	mass (wt %)					
samples	C (%)	O (%)	Mg (%)	Al (%)	Si (%)	Ca (%)
no CO_2_ treatment	39.85	21.87	14.16	1.97	2.64	19.5
CO_2_ soaking time (3 days)	30.64	22.96	11.69	2.36	0.35	32
CO_2_ soaking time (7 days)	27.69	25.78	11.3	2.89	3.85	28.48
CO_2_ soaking time (14 days)	6.65	35.91	3.26	3.55	14.04	36.59

**Figure 14:**
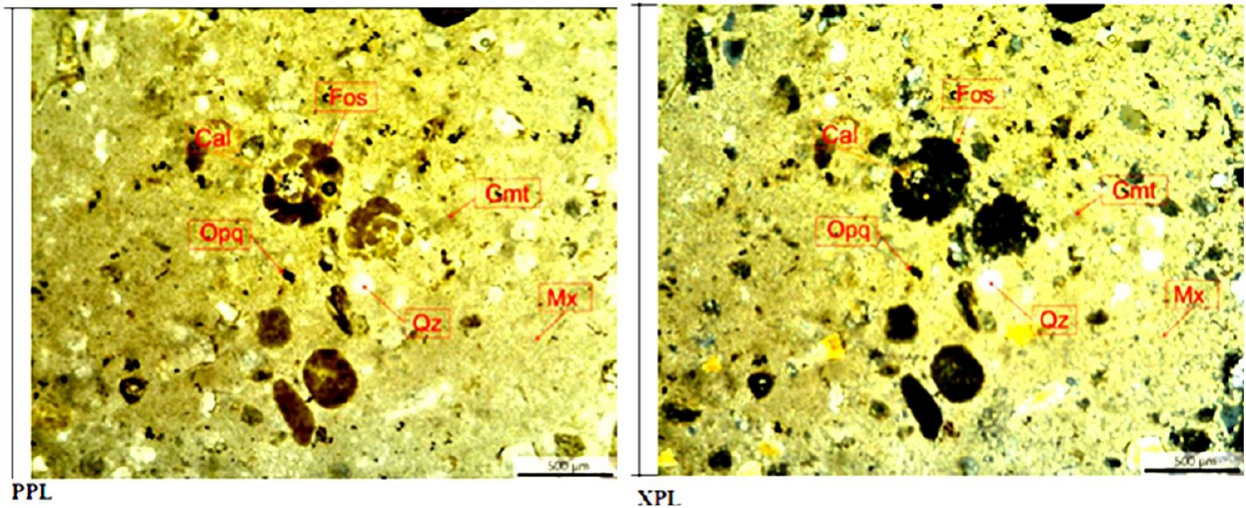
Petrographic thin section of ABF sample with no CO_2_ treatment: Microscopic description of petrographic thin section shows that the appearance of carbonate siltstone with fragments observed in the form of fossil forams (Fos), calcite (Cal), quartz (Qz), and opaque minerals (Opq). The matrices (Mx) were observed to be initially calcite, which has altered into dolomite (rhombus-shaped) due to dolomitization, the cement (Cmt) is observed to be carbonate clay.

The dissolution rate of minerals (e.g., calcite and dolomite) is influenced by solution pH,^19−21^ temperature,^19^ CO_2_ partial pressure, and rock reactive surface area.^22,21,23^ Many researchers have performed experiments on CO_2_-fluid-rock interaction. Shogenov et al.^24^ observed the mineral dissolution (in short term) and precipitation (in long term) by measuring the alteration of effective porosity and permeability, weight of samples, bulk and matrix density, and P and S wave velocities by simplified CO_2_ injection experiment (the samples were placed into Hastelloy cell under vacuum condition and acid solution was injected until 10 bar under temperature of 60 °C) that only considers the impact on rock contact area. Several authors have also investigated geomechanics and reservoir properties alteration due to CO_2_ injection in carbonate^4^ and shale^6,25^ and showed various results depending on rock minerals compositions.

In most cases, sand prediction is performed utilizing analytical and numerical models. Rahmati et al.^26^ reviewed the predictability performance of more than 30 sand prediction analytical and numerical models that have been published worldwide and found that analytical models are quick and easy to use but only suitable for sand onset prediction. However, the impact of rock physics alteration due to CO_2_-water-rock interactions on sanding problem has not been fully investigated yet. Generally, sand prediction models were developed by considering multiphase flow and water production effects.^27−30^ Willson et al.,^31^ Vaziri et al.,^32^ and Palmer et al.^33^ developed a practical and considerable sand onset criteria model based on Critical Bottom Hole Flowing Pressure (CBHFP). The model was based on a rock stress model with shear failure criteria, assuming that rocks are linear elastic. Sand production was assumed to occur at the time when the maximum value of the effective tangential stress around the perforation exceeds the effective strength of the formation rock (in the literature,^31−35^ the strength of the formation rock can be analogized as the unconfined compressive strength (UCS), thick-walled cylinder (TWC), or the empirical function of TWC). The sand onset criterion was given by Willson et al. as follows:^31^
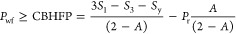
7where *P*_wf_ is the well flowing bottom hole pressure (psi), *S*_1_ and *S*_3_ are the major and minor principal stresses (psi), respectively, *S*_y_ is the effective rock strength (psi), *P*_r_ is the average reservoir pressure (psi), and *A* is the poro-elastic constant as a function of Poisson’s ratio (v) and Biot’s constant (α) as follows:
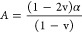
8α is usually assumed as 1.^36^ Dynamic Poisson’s ratio (*v*) can be derived from compressional wave or P wave velocity (*V*_p_) and shear wave or S wave velocity (*V*_s_) as follows:^36^
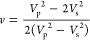
9

Figure 2 shows the illustration of CBHFP sand onset criteria. To prevent sand production, the *P*_wf_ vs *P*_r_ profile should be above the CBHFP line (green area). When the *P*_wf_ vs *P*_r_ profile is below the CBHFP line (red area), then sand production will likely occur.

To the best of the author’s knowledge, research on sand problems due to CO_2_-brine-rock interactions is still limited. This study places special emphasis on rock physics alteration due to CO_2_-brine-rock interaction that hypothetically affecting the sand onset model. Experimental works were conducted to investigate pore-scale mechanisms of mineral dissolution and elastic wave velocity alteration after the rock sample was exposed to CO_2_-brine. Elastic wave velocity indirectly correlates with rock strength, which dominantly affects sand problem in producer well. Several sets of new empirical relations are offered to represent these complex phenomena. Subsequently, this study would observe the impact of rock strength alteration due to CO_2_-brine-rock interactions on sand onset prediction in an arbitrary producer well and would demonstrate that considering CO_2_-brine-rock interactions could help to design a better sand management strategy in producer wells.

## Experimental Methodology

### Material and Sample Preparations

#### Synthetic Brine

Synthetic brine was used to create similar brine salinity properties in Air Benakat Formation (ABF), i.e., 15,000 ppm. To prepare a synthetic brine of 15,000 ppm of NaCl, 30 g of NaCl (chemical pure grade, purity ≥99%, form: white crystalline solids) was dissolved in 2.0 L of demineralized water (Aqua DM, Total Dissolved Solid: 0–3 ppm). The solution was then stirred using a magnetic stirrer for about 15–30 min at 60 rpm to obtain a homogeneous NaCl solution. The pH of the solution was measured using an ATC pH meter (accuracy of ±0.01 pH). The mineral composition of brine (e.g., calcium, magnesium, chloride, sodium, silica) was also observed using standards from the American Public Health Association (APHA),^37^ as shown in Table 3.

#### Core Sample

An outcrop of Air Benakat Formation (ABF) sandstone sample in South Sumatra basin was used in this study (Figure 3). The ABF samples have porosity ranging between 16 and 18% and permeability ranging from 10 to 3000 mD.^38,39^ The samples were taken along the Lematang River in Lahat, South Sumatra, and geological map review has been conducted to ensure the ABF outcrop location as reported by Aziz et al.^5^Table 1 provides the mineralogical composition of the samples according to X-ray diffraction (XRD) measurement. The main mineral in ABF core sample is dolomite (79%) with quartz (17%) and a small amount of clay kaolinite (4%).

For XRD, scanning electron microscopy–energy-dispersive spectroscopy (SEM-EDS), and petrographic thin section measurements purposes, the sample was thinly sliced with approximately 10 mm of length and width, and 5 mm of thickness (Figure 4). The samples were dried using an oven with a temperature of 60 °C and then weighed using a digital mass balance (Fujitsu FSR-A Precision balance with a capacity of 220 g and precision of 0.001 g) periodically.

For P and S wave measurements, the cylindrical sample was used with 38.1 mm of diameter and 55.88 mm of length (Figure 5). To preserve clay mineral conditions, the sample was saturated by synthetic brine following the procedure from McPhee et al.^40^ and API RP40,^41^ i.e., the sample was stored in a sealed tube filled with synthetic brine, then the residual gas saturation was removed using a vacuum pump for about 1–2 h until the gas bubble disappeared, and the sample was saturated for at least 1 day. To avoid bias observation of P and S wave alteration due to CO_2_-brine-rock interaction and to preserve clay-bound water, the free water in rock was removed using centrifugal with an average speed of 2500 rpm (Damon IEC Division HN-S Centrifuge with maximum speed of 4150 rpm) following the procedure reported by Slobod et al.^42^ and Conley and Burrows.^43^ Low centrifuge speed was used to prevent heat and evaporation on core sample. The sample mass was repeatedly weighed using a digital mass balance after it was centrifuged until its mass was not altered. Then, the sample was stored in sealed plastic wrap and could be used for P and S wave measurements.

Analogue well of P-3 from an adjacent oilfield has been selected to represent reservoir conditions of core measurements (Table 2). The depth interval of 895–905 m-TVD was selected since it has similar rock mineral composition (from P-3 cutting samples description report) to our rock sample. Figure 6 shows that the fault regime in ABF interval is strike-slip (*S*_Hmax_ ≥ *S*_V_ ≥ *S*_hmin_). *V*_p_ and *V*_s_ in analogue P-3 depth interval are also in range with core measurement in this study (Figure 18).

### Methods

#### CO_2_-Brine-Rock Batch Experiment

The schematic and procedure of CO_2_-brine-rock batch experiment used in this study are shown in Figure 7.

#### X-ray Diffraction (XRD), Scanning Electron Microscopy–Energy-Dispersive Spectroscopy (SEM-EDS), and Petrographic Thin Section

Sample mineralogy was measured quantitively using a Rigaku SmartLab X-ray diffractometer (XRD). The samples were prepared in powder with 200 mesh size.

SEM-EDS was measured using JEOL JSM 6510 LA to characterize rock morphology and to map rock elements quantitatively. A petrographic thin section was observed using a polarized microscope to identify rock mineralogy and texture.

#### P and S Wave Measurement and Porosity Measurement

The core was prepared in cylinder form following standard procedures from ASTM D4543.^44^ P and S wave measurements were conducted by using pressurized rock physics measurement tools SeisCore.^45^ All measurements were conducted at room temperature (25 ± 3 °C, depending on weather conditions).

Core sample porosity was measured by using a gas porosimeter (PORG 100) at atmospheric conditions. The core sample was dried by using an oven at 50 °C to prevent clay and mineral damages. Porosity measurements were only conducted at the initial (before CO_2_ treatment) and final (after being soaked by CO_2_ for 18 days) stages to prevent core damage due to multiple treatments and preparations.

#### Sand Onset Modeling

The sand onset criteria used in this study were given by Willson et al.,^31^ as shown in eq 7. Several authors have formulated empirical equations for estimating minimum stress *S*_hmin_.^46−51^ In this study, we used Eaton’s correlation to estimate *S*_hmin_ as a function of Poisson’s ratio (*v*) as follows:^48^
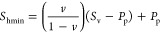
10Jaeger and Cook developed the relation between effective stress, pore pressure, and friction coefficient as follows:^52^
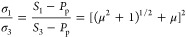
11where μ is the friction coefficient. Byerlee performed laboratory experiments about μ for different spectrum of rock types and concluded that μ ranged between 0.6 and 1.0 (0.6 ≤ μ ≤ 1.0).^53^ Jaeger and Cook^52^ stated that the typical value of rock friction coefficient μ is 0.6. If we use μ = 0.6 in eq 11, then we obtain^52^
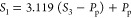
12

From Table 2, it was known that faults regime in ABF was strike-slip. Thus, we obtained that *S*_1_ is *S*_Hmax_, *S*_3_ is *S*_hmin_, and *S*_2_ is *S*_v_. Since we have limited core samples, we used empirical correlations to estimate rock strength properties, i.e., UCS and TWC. Chang et al. summarized 31 empirical correlations between UCS and physical rock properties (such as elastic velocity, modulus, and porosity)^54^ for sandstone,^55−57^ limestone and dolomite,^58,59^ and shale.^60,61^ Since ABF core sample is dominated by dolomite, we selected Golubev and Rabinovich correlation for dolomite or limestone to estimate UCS as follows:^58^
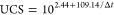
13where UCS is unconfined compressive strength (in MPa) and Δ*t* is  (in μs/ft).

In addition, Khaksar et al. summarized more than 20 empirical correlations of TWC and UCS or porosity.^62^ We selected the Rahman et al. correlation, which represents tertiary weak/intermediate rocks in Southeast Asia as follows:^63^
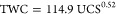
14This TWC value would be used later as *S*_y_ in eq 7. Since there is a possibility that UCS and TWC values would have specific values for different locations, we suggest using experimental laboratory data results if sufficient core samples are available.

#### Experimental and Interpretation Uncertainties

The uncertainties in results interpretation were possibly caused by a limited number of core samples available for experiments. XRD, SEM-EDS, and petrographic thin section results still also have uncertainties regarding the heterogeneity of rock samples acquisition even though samples were taken from nearby locations.

In dry mass measurements, there was a possibility of errors due to evaporation of water saturation caused by heat from the centrifuge process. The error of acoustic velocities measurements was ± 5% due to uncertainties in picking first arrival time and length-to-diameter ratio less than 2. The error of porosity measurements by using a gas porosimeter (PORG 100) was ±0.3%. The error of pH measurements was less than 3%.

All the experimental works were conducted at room temperature, i.e., 27 ± 3 °C. Spycher et al.^12^ showed that temperature decreases cause an increase in CO_2_ solubility in water, and Kumar et al.’s simulation works showed that temperature did not affect pH and porosity significantly in dolomite rocks.^13^

The assumption of Willson et al.’s sand onset prediction model was based on a rock stress model with only considering shear failure criteria, assuming that rocks are linear elastic. This model did not consider friction effect due to fluid flow, effect of hydrocarbons and impurities, and anisotropy of rock samples. The uncertainties were also quite high in sand onset prediction since we used empirical correlations and assumptions to estimate rock strength parameters (i.e., UCS, TWC, friction coefficient, and Biot’s constant) due to limited core samples. We suggest using experimental laboratory data to measure and to validate rock strength if core samples are available.

## Results and Discussion

### Brine Analysis

Brine composition before and after CO_2_ treatments was measured to observe rock mineral dissolution that dissolved into brine. Figure 8 shows the brine samples that have been analyzed in this study. In Figure 8b, CO_2_ bubbles are observed dissolving into brine samples after being injected with CO_2_ for 3 days. Figure 9 shows brine pH profiles that were recorded 3–4 times per day. Brine pH decreased ∼22% after it was exposed to pH of more than 8 h. This result is aligned with previous reported experimental studies.^64−66^Table 3 shows a comparison of brine compositions before and after CO_2_ injection. Calcium was observed to increase (from 24.3 to 103.0 mg/L), which indicates dolomite dissolution, while magnesium showed a smaller increase (from 6.60 to 7.04 mg/L). It can be explained that calcium is more reactive than magnesium because calcium has larger atomic radius and greater distance between its nucleus and valence electrons. It makes calcium easier to lose electrons and more reactive than magnesium. In addition, a smaller increase of magnesium can possibly be explained by secondary magnesium-bearing minerals precipitation (e.g., magnesite (MgCO_3_)) due to CO_2_ injection.^67,68^ Unfortunately, a thorough analysis is not conducted in this study to confirm this phenomenon, and we suggest conducting further research. Sodium, silica, and aluminum were observed to remain at similar magnitudes, which implies that kaolinite did not significantly react with carbonic acid. Chloride was observed to decrease after being exposed to CO_2_. Several studies indicated salt precipitation phenomena due to CO_2_ injection in dolomite.^69−72^

### Dry Mass

Dry mass measurements were measured after core samples were exposed to CO_2_-brine and centrifuged as shown in Figure 10. It was observed that dry mass decreased ∼1.4% after it was exposed to CO_2_-brine over time, which indicates mineral dissolution phenomena.

### XRD

XRD measurements were conducted for cube samples with various CO_2_ soaking times (0–14 days), as shown in Figure 11. In general, there were no new peaks of 2 theta, showing no mineralization occurred. Figure 12 shows the indication of dolomite dissolution (black dotted circle) as reduction in dolomite intensity peaks.

XRD quantitative analysis (Table 4) clearly shows that dolomite concentration is decreasing over CO_2_ soaking time. These results indicate that dolomite dissolution phenomena occurred when rock samples interacted with carbonic acid.

### SEM-EDS

In general, SEM analysis shows that ABF rock samples consist of intercrystalline porosity which typically describes dolomite crystal (green square in Figure 13). After rock samples were soaked by CO_2_ (3–14 days), there were indications of dissolution or secondary porosity development (red square in in Figure 13).

EDS analysis (Table 5) shows that magnesium (Mg) element mass (wt %) is decreasing over CO_2_ soaking time, which indicates dolomite dissolution. The increase in calcium (Ca) element could be possibly due to the indication of calcium-bearing minerals precipitation (e.g., calcium carbonate (CaCO_3_)^73^), and the source of calcium for this mineral precipitation might be the dissolved calcium.

### Petrographic Thin Section

**Figure 15:**
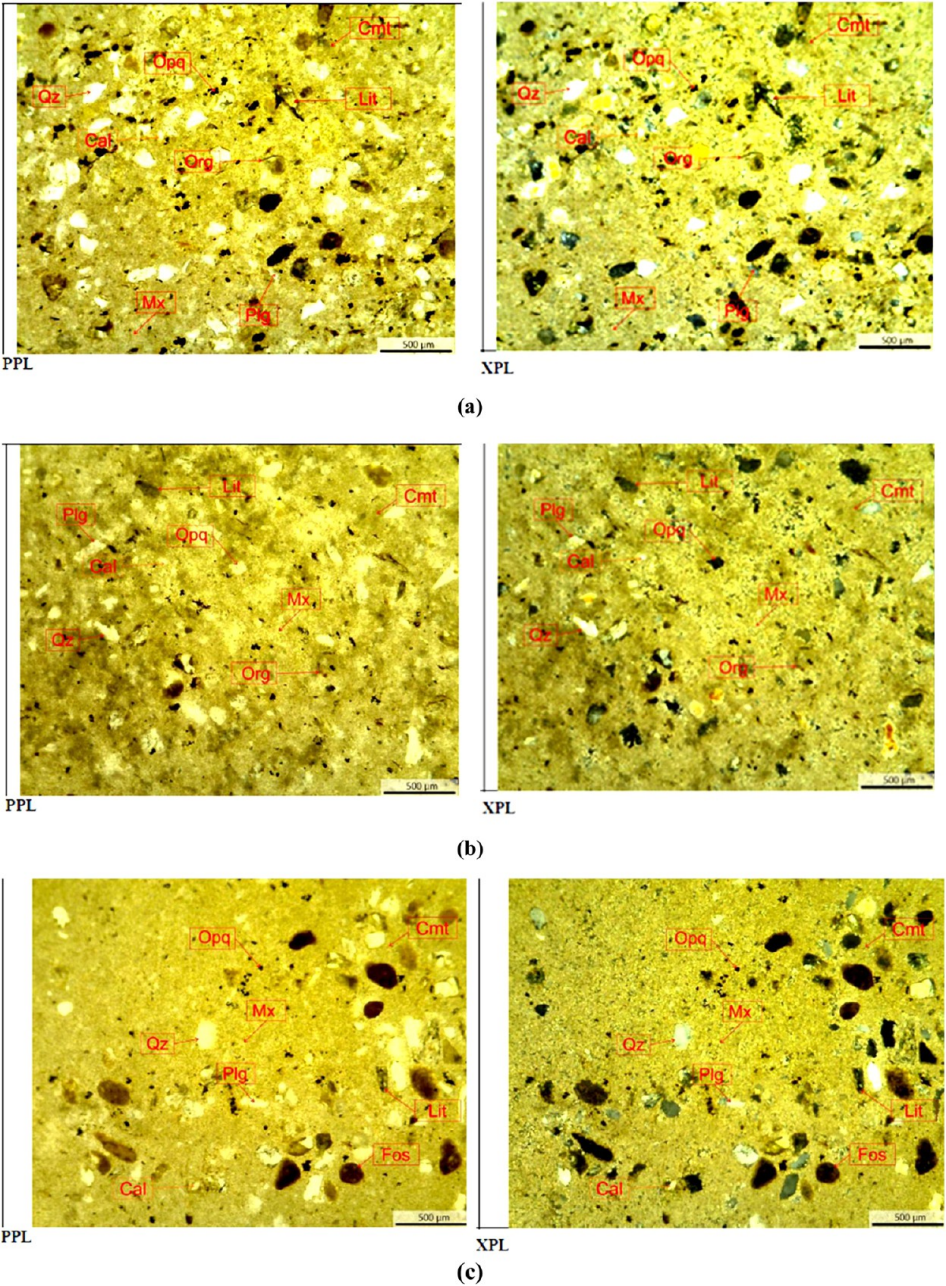
Petrographic thin section of ABF samples with CO_2_ treatment: (a) soaking of 3 days; (b) soaking of 7 days; (c) soaking of 14 days.

Petrographic thin section analysis has also been conducted on ABF rock samples before and after CO_2_ treatment (Figures 14 and 15). In general, ABF rock samples consist of quartz, opaque mineral, and matrices as calcite and dolomite, which appear in fragment, and cement as carbonate clay, which appears in matrices. Quantitative mineral analysis has been conducted on thin sections of ABF samples as shown in Table 6. The tendency of calcite and cement (carbonate) reduction was observed after ABF rock samples were soaked by CO_2_ which indicates mineral dissolution.

**Table 6:** Mineralogical Composition of ABF Samples before and after CO_2_ Treatment from Petrographic Thin Section

	composition (wt %)					
thin section samples	quartz (%)	calcite (%)	matrices (%)	cement (%)	organic materials (%)	opaque minerals (%)
no CO_2_ treatment	13	2	45	15	15	10
CO_2_ soaking time (3 days)	12	1	45	10	10	7
CO_2_ soaking time (7 days)	10	1	65	10	4	3
CO_2_ soaking time (14 days)	10	1	65	5	5	3

### Porosity Measurements

Cylindrical sample porosity measurements have been conducted at the initial (before CO_2_ treatment) and final (after being soaked by CO_2_ for 18 days) conditions as shown in Table 7. It shows that porosity increased ∼6.6% after it was soaked by CO_2_-brine for 18 days. These results are aligned with recent reported experimental studies.^74−80^ For longer-term behavior of porosity changes, Kumar et al. (2020) have reported from geochemical simulations of dolomite layer for 50 years of CO_2_ injection simulation that porosity alteration magnitude due to CO_2_ injection was insignificant (i.e., only about 0.1%).^16^

**Table 7:** Porosity Measurements before and after CO_2_ Treatment (Being Soaked for 18 Days)

condition	porosity (%)
no CO_2_ treatment	12.63
CO_2_ soaking time (18 days)	13.46

### P and S Wave (*V*_p_ and *V*_s_)

**Figure 16:**
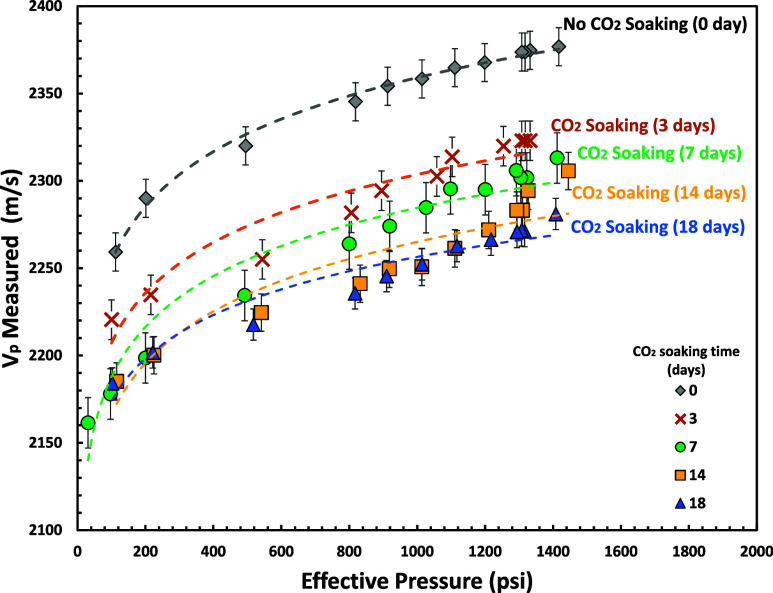
*V*_p_ measurements for various effective pressures and CO_2_ soaking times (0–18 days) using SeisCore.

**Figure 17:**
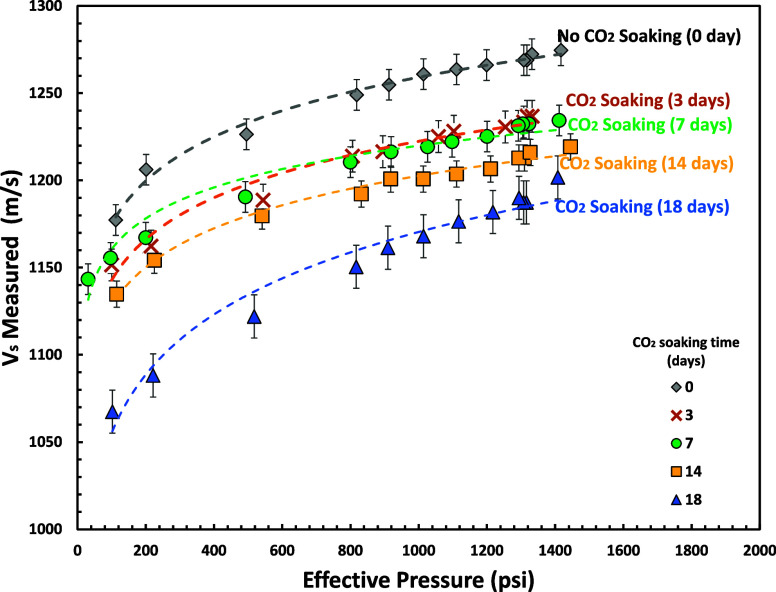
*V*_s_ measurements for various effective pressures and CO_2_ soaking times (0–18 days) using SeisCore.

*V*_p_ and *V*_s_ measurements for various effective pressure and CO_2_ treatment/soaking time have been demonstrated by using SeisCore (shown in Figures 16 and 17). Our experimental results show a nonlinear (power-law) relationship between elastic velocity and effective pressure. These results are similar to previously reported experimental studies.^81−90^

This study shows that *V*_p_ and *V*_s_ were decreased after 18 days of CO_2_-brine soaking time (∼4.3% *V*_p_ and ∼6.2% *V*_s_ reduction at 1300 psi of effective pressure) which complied with reported experimental studies.^91,92^

**Figure 18:**
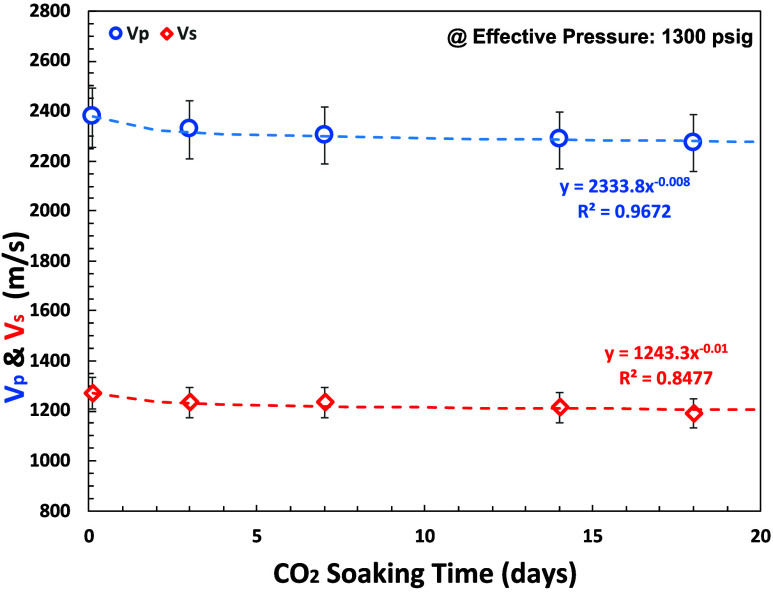
*V*_p_ and *V*_s_ measurements for various CO_2_ soaking times (0–18 days) at effective pressure = 1300 psi (benchmark to ABF interval in P-3 as analogue well) using SeisCore.

**Figure 19:**
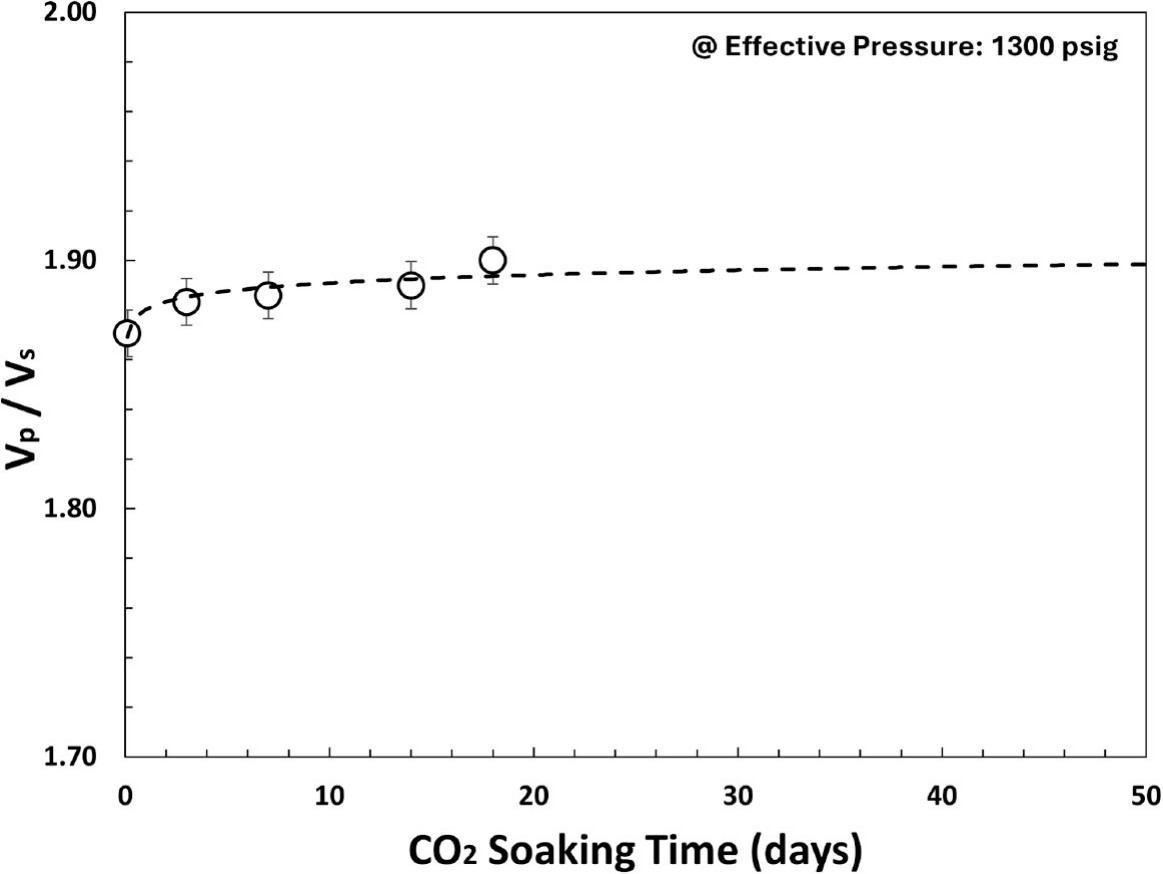
*V*_p_/*V*_s_ for various CO_2_ soaking times (0–18 days) at effective pressure = 1300 psi (benchmark to ABF interval in P-3 as analogue well) using SeisCore.

**Figure 20:**
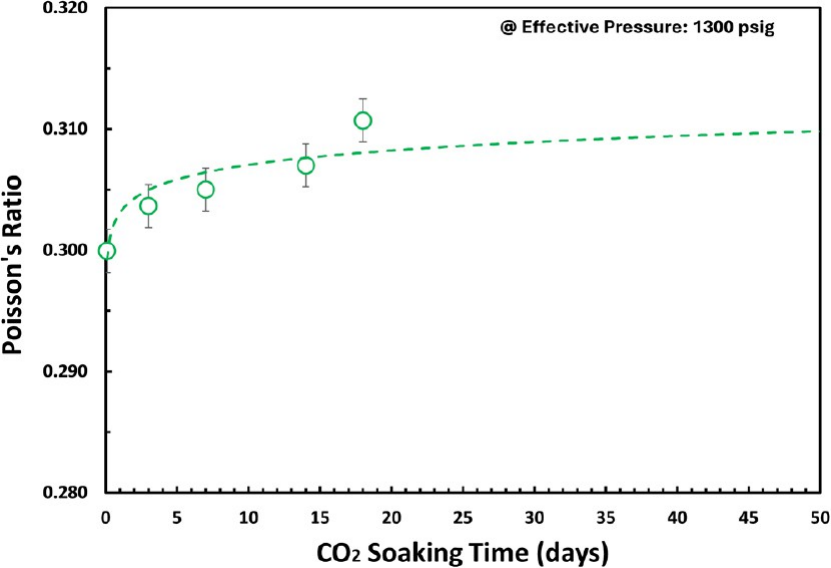
Poisson’s ratio for various CO_2_ soaking times (0–18 days) at effective pressure = 1300 psi (benchmark to ABF interval in P-3 as analogue well) using SeisCore.

Our experimental study demonstrates that *V*_p_, *V*_s_, *V*_p_/*V*_s_, and Poisson’s ratio have power-law relationship with pressure and CO_2_ soaking time as shown in Figures 18, 19, and 20. Indirectly, it demonstrates rock strength reduction due to CO_2_-brine interaction.

We have constructed an empirical relationship between *V*_p_ and *V*_s_ and pressure and CO_2_ soaking time. If the rock sample was not soaked with CO_2_ (dry), then
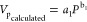
15
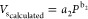
16and if rock samples were soaked with CO_2_ (*t* > 0 day), then
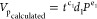
17
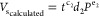
18where *V*_p_calculated__ is the calculated P wave (m/s), *V*_s_calculated__ is the calculated S wave (m/s), *t* is the CO_2_ soaking time (days), *P* is the effective pressure (psi), and *a*, *b*, *c*, *d*, and *e* are the empirical constants.

We used nonlinear regression (generalized reduced gradient or GRG) method to obtain empirical constant values that result minimum error. Error functions that we used in this study are root-mean-square error (RMSE), relative RMSE, and *R*^2^ as follows:
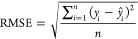
19
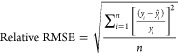
20
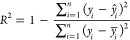
21where *y*_*i*_ is the measured value, *ŷ*_*i*_ is the predicted value, *y̅*_*i*_ is the mean value of observations data, and *n* is the number of observations.

**Figure 21:**
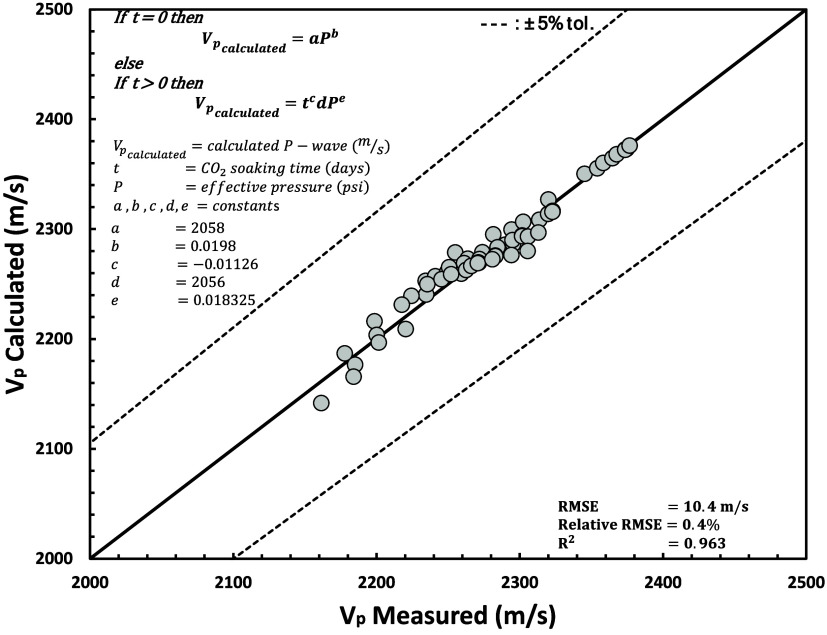
*V*_p_ measured and calculated using nonlinear regression using eqs 15 and 17.

**Figure 22:**
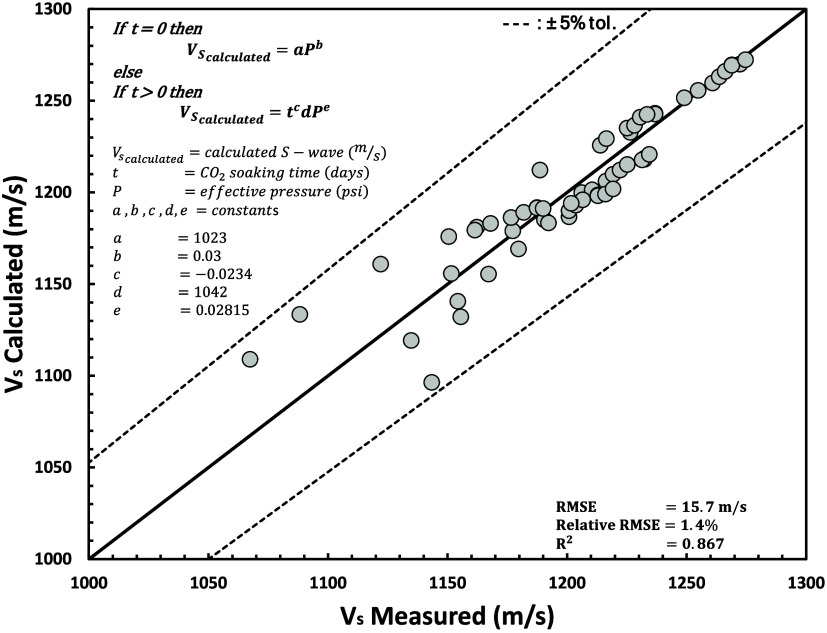
*V*_s_ measured and calculated using nonlinear regression using eqs 16 and 18.

Figures 21 and 22 show the results of nonlinear regression between measured and calculated values of *V*_p_ and *V*_s_, which result in relatively good correlations (shown by low RMSE and relative RMSE and high *R*^2^) and can be used to predict *V*_p_ and *V*_s_. For predicted *V*_s_, correlations are relatively lower in low *V*_s_ and effective pressure but still in the range of ±5% tolerances.

### Time-Dependent Sand Onset Modeling of CO_2_-Brine-Sandstone

#### Synthetic 3D Reservoir Model

**Figure 23:**
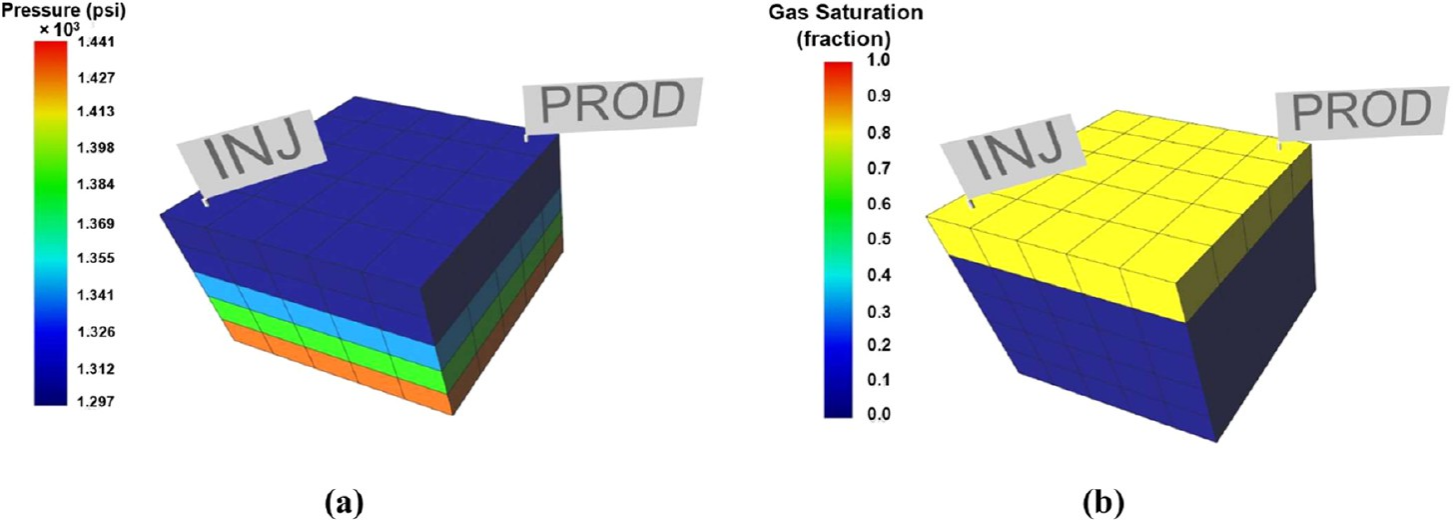
Synthetic homogeneous gas reservoir model with one producer and injector: (a) reservoir pressure (psi); (b) initial gas saturation.

In this section, we used a simple homogeneous synthetic gas reservoir model to generate well bottom hole pressure and pore pressure (or reservoir pressure). We used a compositional numerical model (using Peng–Robinson equation of state) with one producer and injector that operate simultaneously. Detailed reservoir model properties are shown in Table 8 and Figure 23.

**Table 8:** Properties of Synthetic Gas Reservoir Model

parameter	unit	value	parameter	unit	value
model size (*L*_*x*_ × *L*_*y*_ × *L*_*z*_)	m	762 × 762 × 152	Corey exponent		2.0
model dimension (*N*_*x*_ × *N*_*y*_ × *N*_*z*_)		5 × 5 × 5	initial gas saturation (*S*_gi_)		0.8
grid top	m	884	irreducible water saturation (*S*_wirr_)		0.2
gas water contact (GWC)	m	930	residual gas saturation (*S*_gr_)		0.1
initial reservoir pressure	psi	1300	max. gas relative permeability (*k*_rg_)		0.7
reservoir temperature	°F	158	max. water relative permeability (*k*_rw_)		0.7
porosity		0.2	total pore volume	res.ft^3^	6.275 × 10^8^
horizontal permeability (*k*_H_)	mD	48	total hydrocarbon pore volume	res.ft^3^	1.004 × 10^8^
anisotropy (*k*_V_/*k*_H_)		0.1	original gas in place (OGIP)	std.ft^3^	8.270 × 10^9^
rock compressibility (*c*_r_)	1/psi	3 × 10^–6^	CO_2_ injection rate	std.ft^3^	3.00 × 10^6^
water compressibility (*c*_w_)	1/psi	2 × 10^–6^	gas production rate	std.ft^3^	3.00 × 10^6^
CH_4_ composition	%	100	maximum injector bottom hole pressure (BHP)	psi	1652
initial gas formation volume factor (*B*_gi_)	res.ft^3^/std.ft^3^	0.0121			

**Figure 24:**
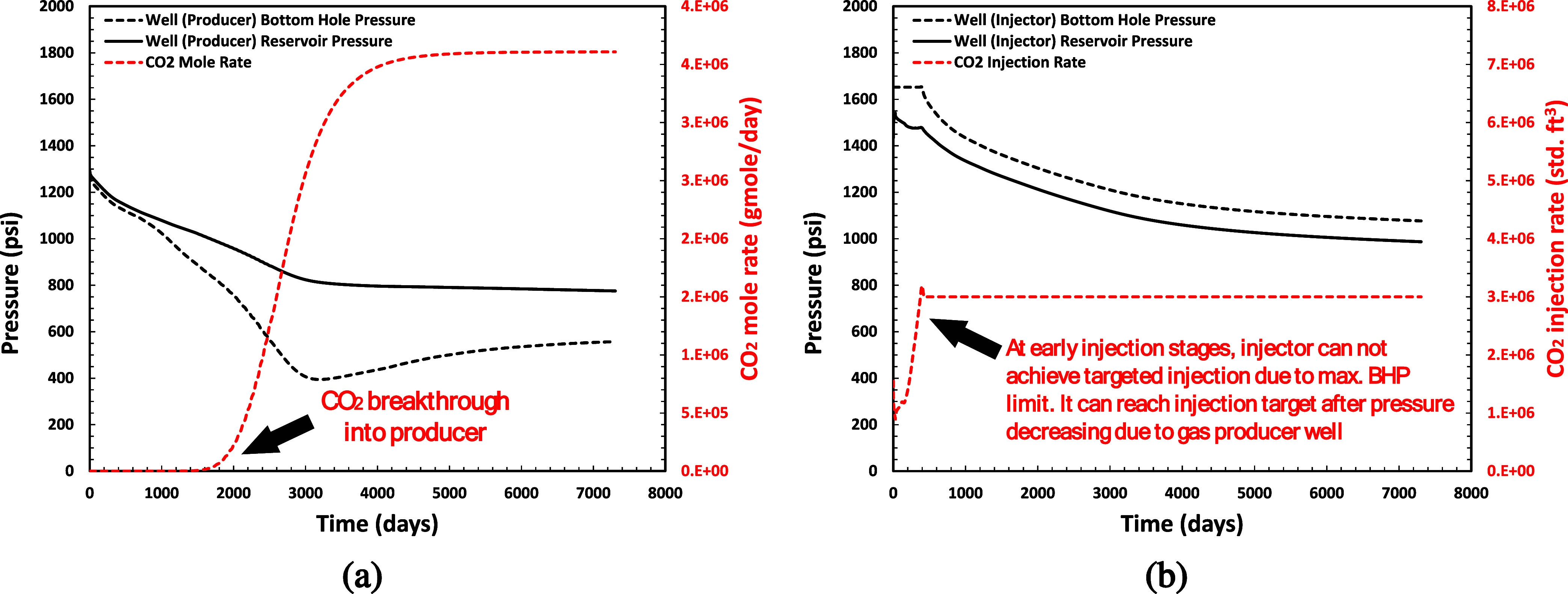
Simulation results for each well: (a) producer; (b) injector.

The results of simulation reservoir are shown in Figure 24. Figure 24a shows CO_2_ breakthrough into producer well at *t* ≈ 850 days (depicted in Figure 24a as the CO_2_ mole rate profile increases). At early injection stages (Figure 24b), the injector cannot achieve targeted injection due to the maximum BHP limit. It can reach the injection target after pressure decreases due to gas producer well. These results will be utilized as a case study for sand onset modeling in a later section.

#### Sand Onset Modeling

In this section, we utilized reservoir simulation results (i.e., reservoir pressure and BHP) as an input for sand onset modeling. We investigated 2 cases of CBHFP to see the impact of CO_2_-brine-rock interaction, i.e., case without and with CO_2_-brine-interactions.

**Figure 25:**
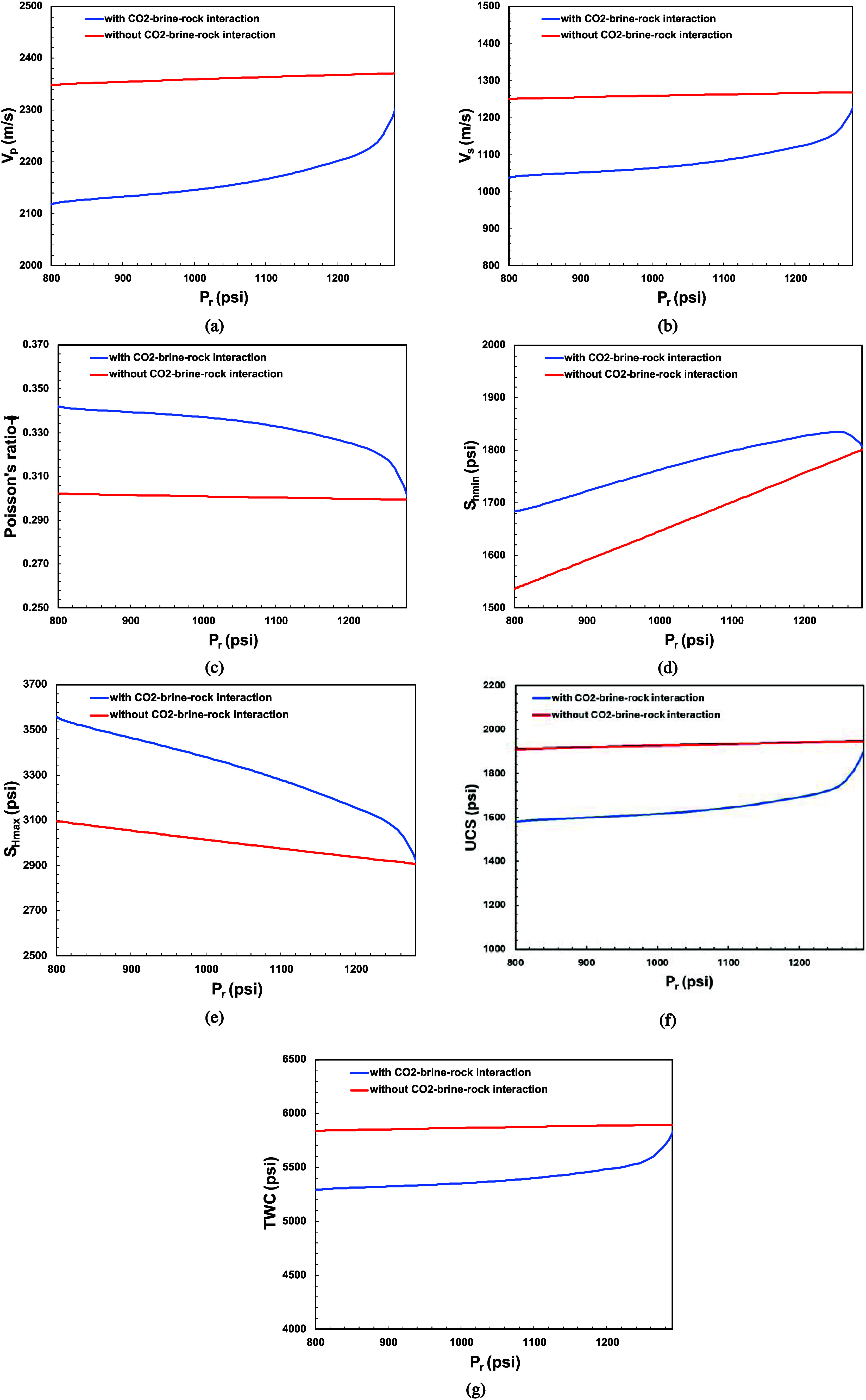
Sand model parameters estimation vs pressure for all cases: (a) *V*_p_; (b) *V*_s_; (c) Poisson’s ratio; (d) *S*_hmin_; (e) *S*_Hmax_; (f) UCS; (g) TWC.

Each case has different *V*_p_, *V*_s_, *v*, *S*_hmin_, *S*_Hmax_, UCS, and TWC or *S*_y_ profile over pressure generated from reservoir simulation (Figure 25). *V*_p_ and *V*_s_ profiles (Figure 25a,b) decrease as reservoir pressure decreases. However, the decrease of *V*_p_ and *V*_s_ profile in the case of CO_2_-brine-rock interaction (blue line) is sharper following power-law relationship as it integrates CO_2_-brine contact time with reservoir rock. Poisson’s ratio profile (Figure 25c) increases as reservoir pressure decreases, which aligns with Yu et al.’s (2016) study as it correlates with porosity increases and elastic moduli decreases in sandstone.^93^ Porosity improvement is correlated with dolomite dissolution as rock was contacted with carbonic acid. UCS and TWC profiles (Figure 25f,g) decrease as reservoir pressure decreases. The decrease in the case of CO_2_-brine-rock interaction (blue line) shows that the presence of CO_2_-brine-rock interactions weakens the rock strength compared to without CO_2_-brine-rock interaction (red line).

**Figure 26:**
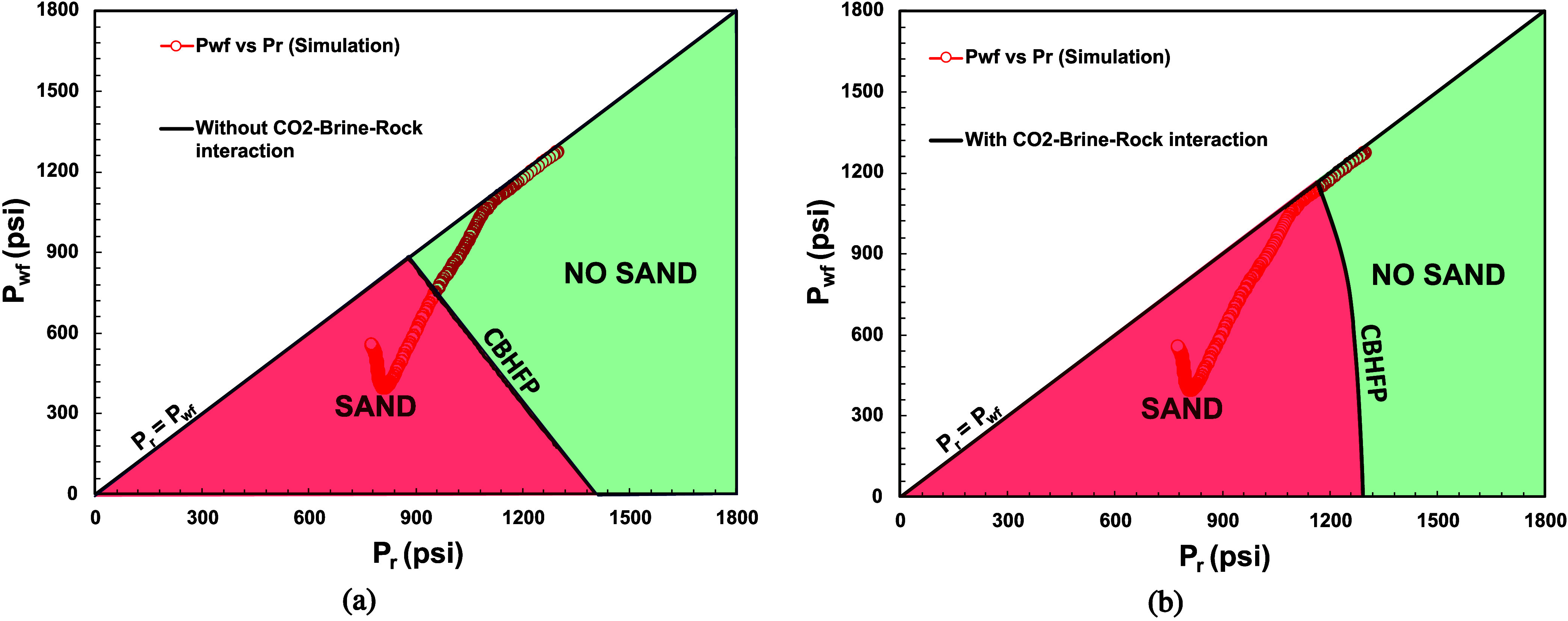
Sand onset CBHFP modeling for case: (a) without CO_2_-brine-rock interaction; (b) with CO_2_-brine-rock interaction.

**Figure 27:**
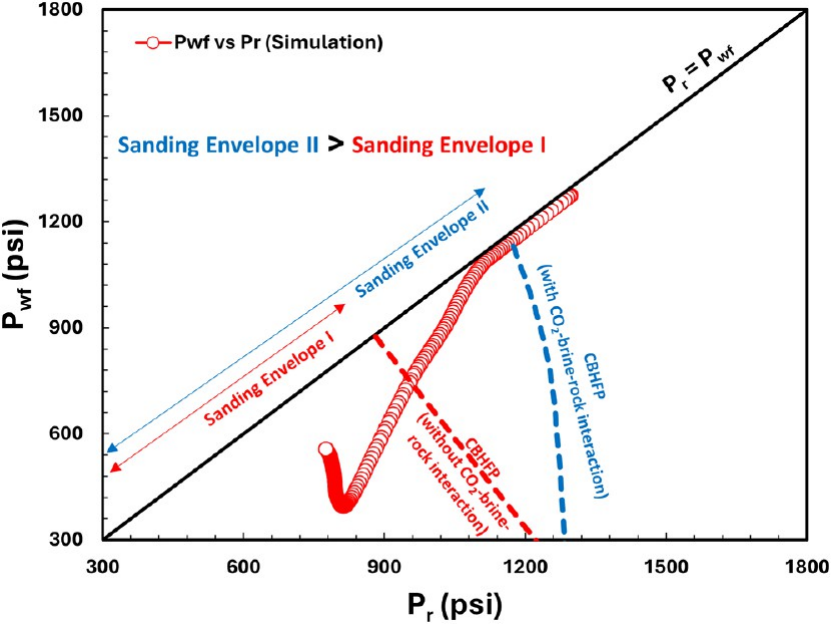
Overlay sand onset CBHFP modeling for the case without CO_2_-brine-rock interaction (red dashed line) and with CO_2_-brine-rock interaction (blue dashed line).

Subsequently, the above parameters were used as input for eq 7. Figure 26 depicts the results of sand onset (sand-free envelope) CBHFP models for both cases. Sand onset in the case of CO_2_-brine-rock interaction (Figure 26b) occurred faster (when *P*_r_ < ∼ 1175 psi and *P*_wf_ < 1140 psi) than without CO_2_-brine-rock interaction (when *P*_r_ < ∼950 psi and *P*_wf_ < 750 psi). Figure 27 depicts the comparison of CBHFP profiles for both cases. It clearly shows that CO_2_-brine-rock interaction consideration on sand onset modeling makes production well more prone to sand problem and can affect sand problem management strategy.

## Conclusions

This study demonstrates experimental works to observe CO_2_-brine-rock interaction phenomena in CO_2_ injection in dolomite-rich sandstone at Air Benakat formation (ABF) by using CO_2_-brine-rock batch experiment setup. Brine composition analysis, dry mass measurements, XRD, SEM-EDS, petrographic thin sections analysis, and porosity measurements show indications of dolomite dissolution that leads to porosity improvement up to ∼6.6%. Salt precipitation was suspected from chloride reduction by brine composition analysis, but this evidence needs to be supported by further experimental works.

Elastic wave velocity measurements for various CO_2_ soaking times and effective pressures resulting in *V*_p_ and *V*_s_ reduction (∼4.3% of *V*_p_ and ∼6.2% of *V*_s_ reduction at 1300 psi of effective pressure) that indirectly implies rock strength reduction due to CO_2_-brine-rock interactions. These results comply with porosity improvement.

We developed an empirical correlation to estimate *V*_p_ and *V*_s_ as a function of CO_2_ soaking time and effective pressure that have relatively good correlations with measured data. Since we have limited core samples, we used empirical correlations from previously reported studies to estimate rock strength properties that were used as an input for sand onset prediction model. We suggest using experimental laboratory data results if sufficient core samples are available to obtain more reliable UCS and TWC values. This correlation is used to construct modification of sand onset model and shows that CBHFP will have different gradient after CO_2_ exposing sandface around the wellbore. Thus, this study shows that CO_2_-brine-rock interactions could potentially accelerate sanding problem in producer wells and can be used to design better sand management strategies in producer wells in CCUS operation.

## Supporting Information Available

*V*_p_ and *V*_s_ measurements as a function of CO_2_ soaking time and effective pressure as shown in Figure 16 and Figure 17 (Table S1) (XLSX)
